# Rice stripe virus p2 protein interacts with ATG5 and is targeted for degradation by autophagy

**DOI:** 10.3389/fmicb.2023.1191403

**Published:** 2023-04-28

**Authors:** Xiangxiang Zhang, Qionglian Wan, Penghuan Rui, Yuwen Lu, Zongtao Sun, Jianping Chen, Yunyue Wang, Fei Yan

**Affiliations:** ^1^Plant Protection College, Yunnan Agricultural University, Kunming, China; ^2^State Key Laboratory for Managing Biotic and Chemical Threats to the Quality and Safety of Agro-products, Institute of Plant Virology, Ningbo University, Ningbo, China; ^3^Key Laboratory of Biotechnology in Plant Protection of MARA and Zhejiang Province, Institute of Plant Virology, Ningbo University, Ningbo, China

**Keywords:** autophagy, rice stripe virus, ATG5, p2, interaction, degradation, eIF4A

## Abstract

Autophagy can be induced by viral infection and plays antiviral roles in plants, but the underlying mechanism is not well understood. In our previous reports, we have demonstrated that the plant ATG5 plays an essential role in activating autophagy in rice stripe virus (RSV)-infected plants. We also showed that eIF4A, a negative factor of autophagy, interacts with and inhibits ATG5. We here found that RSV p2 protein interacts with ATG5 and can be targeted by autophagy for degradation. Expression of p2 protein induced autophagy and p2 protein was shown to interfere with the interaction between ATG5 and eIF4A, while eIF4A had no effect on the interaction between ATG5 and p2. These results indicate an additional information on the induction of autophagy in RSV-infected plants.

## Introduction

Autophagy is a conserved vacuole or lysosome-mediated degradation pathway for clearing and recycling cellular components including cytosol, macromolecules, and dysfunctional organelles (He and Klionsky, 2009). Proteins encoded by autophagy-related genes (*ATGs*) are the key components in the process, which is conserved among mammals, yeast and plants. Increasing evidence demonstrates that autophagy plays key roles in plant defense against viruses (Liu et al., 2005; Hofius et al., 2017; Leary et al., 2019; Yang et al., 2020; Ismayil et al., 2020b). Recently, viral proteins from different viruses have been identified to directly interact with ATGs or other components of the autophagy machine and thus be targeted for autophagic degradation. The capsid protein (CP) of cauliflower mosaic virus (CaMV), the NIb protein of turnip mosaic virus (TuMV) and the virulence factor βC1 of cotton leaf curl Multan virus (CLCuMuV) have been reported to interact with the autophagy cargo receptor NEIGHBOR OF BREAST CANCER 1 (NBR1), ATG6 (also called Beclin1) and ATG8, respectively and to be targeted by autophagy for degradation (Hafren et al., 2017; Haxim et al., 2017; Hafren et al., 2018; Li et al., 2018). In addition, protein 2b from cucumber mosaic virus (CMV) was thought to be degraded by autophagy through the calmodulin-like protein rgs-CaM (Nakahara et al., 2012). Interestingly, the HC-Pro protein of TuMV (as well as its NIb) could interact with NBR1 and similarly be targeted for degradation, indicating that multiple proteins from a single virus might be targeted by autophagy for degradation (Hafren et al., 2018; Li et al., 2018).

Rice stripe virus (RSV), a member of the genus *Tenuivirus* in the family *Phenuiviridae*, is transmitted by the small brown planthopper (*Laodelphax striatellus*) and causes epidemics in rice throughout East Asia (Xiong et al., 2008; Zhang et al., 2012). The RSV genome consists of four single-stranded, negative sense (ambisense) RNA molecules encoding proteins that include an RNA-dependent RNA polymerase (RdRP), p2 (a weak RNA silencing suppressor), pc2 (putative membrane glycoprotein), p3 (RNA silencing suppressor), pc3 (a nucleocapsid protein, NCP), p4, a nonstructural disease specific protein (SP) and pc4, a movement protein (MP; Xiong et al., 2008, 2009; Shen et al., 2010; Wu et al., 2013; Rong et al., 2014; Xu et al., 2021). p2 interacts with SGS3 and also promotes virus systemic movement by interacting with fibrillarin (Du et al., 2011; Zheng et al., 2015). Recently, it has been reported that p2 interacts with OsARF17 and OsMYCs, and impedes their antiviral defense to promote virus infection (Zhang et al., 2020; Li L. et al., 2021).

In a previous report, we demonstrated that the activated autophagy in RSV-infected plants is a defense response against the virus that targets p3 for degradation via a new cargo receptor, P3IP (Jiang et al., 2021). Moreover, a virus-derived small interfering RNA (vsiRNA) that regulates eIF4A, a negative factor of autophagy by interacting with NbATG5 and inhibiting its roles in autophagy, was identified recently to be essential for activating autophagy in RSV-infected plants (Zhang et al., 2021b). We now report that RSV p2 interacts with ATG5 and can also be targeted by autophagy for degradation. Moreover, ATG5 had a stronger affinity for p2 than for eIF4A, suggesting a mechanism of plant defense in which RSV p2 is recognized and ATG5 is released from its interaction with eIF4A to induce antiviral autophagy.

## Results

### *Nicotiana benthamiana* ATG5 interacts with RSV p2 protein

**Figure 1:**
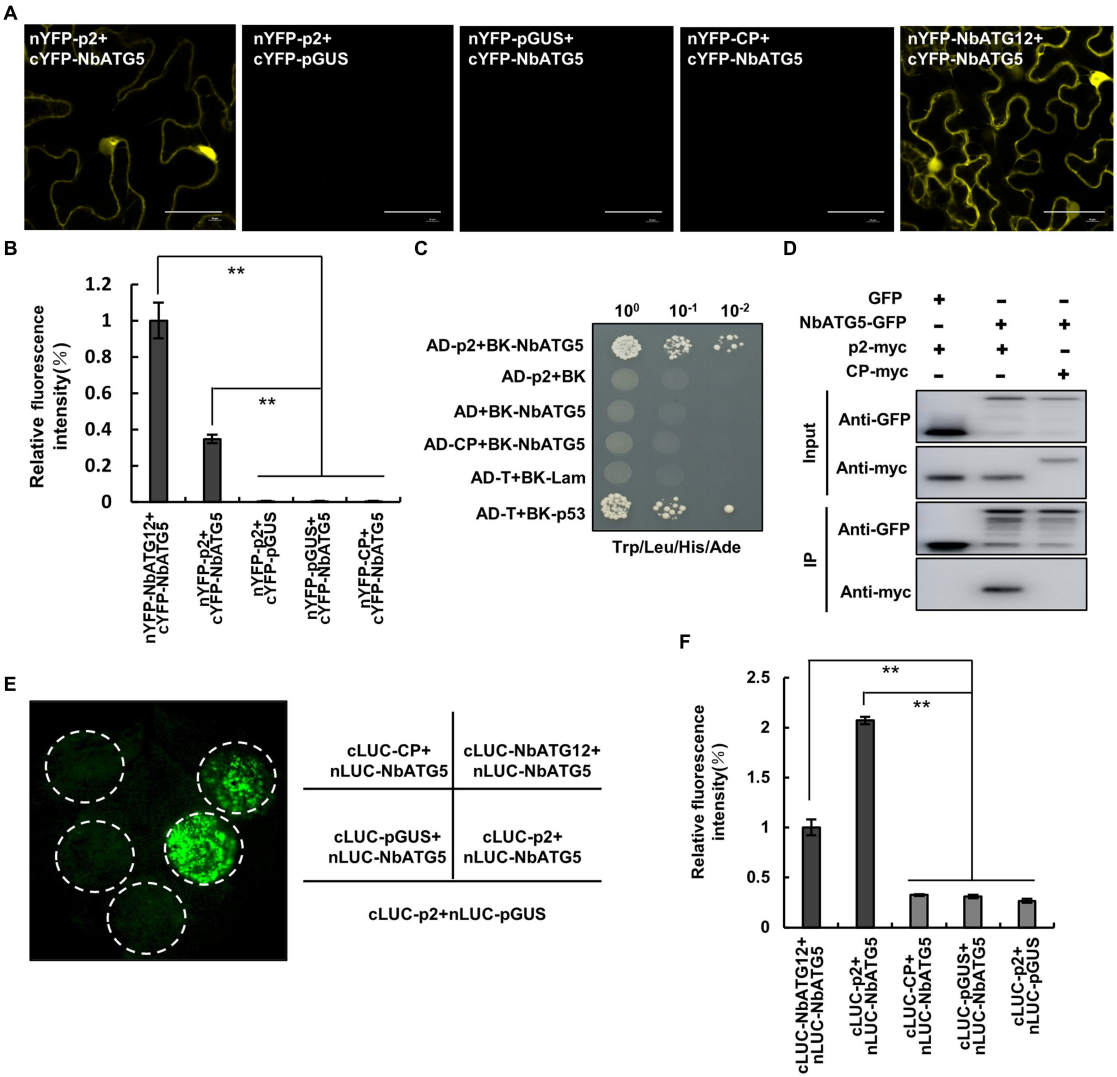
*Nicotiana benthamiana* ATG5 (NbATG5) interacts with RSV p2 protein. **(A)** Images from confocal microscopy showing the BiFC assay for interaction between NbATG5 and RSV p2. Cells were expressing nYFP-p2 and cYFP-NbATG5, and in the controls nYFP-GUS and cYFP-NbATG5, nYFP-p2 and cYFP-GUS, or nYFP-CP and cYFP-NbATG5. nYFP-NbATG12 and cYFP-NbATG5 were expressed in cells as positive controls. Bars, 20 μm. **(B)** Analysis of the intensity of fluorescence in BiFC assay. Bars indicate standard error from three individual experiments. The asterisks indicate significant difference by Student’s *t* test (^**^p < 0.01). **(C)** NbATG5 interacts with RSV p2 protein in a yeast two hybrid (Y2H) assay. Yeast co-transformed with pGAD-T (AD-T) and pGBK-p53 (BK-p53) served as a positive control, and yeast co-transformed with vectors AD-T and pGBK-Lam (BK-Lam), pGAD-p2 (AD-p2) and BK-Lam or AD-T and pGBK-NbATG5 (BK-NbATG5) were the negative controls. Serial 10-fold dilutions of co-transformed yeast cells on synthetic defined (SD) medium (−Ade/–His/−Leu/−Trp) with X-gal are shown. **(D)** Co-IP analysis of the interaction between NbATG5 and p2. NbATG5-GFP, GFP, RSV CP-myc and p2-myc were transiently expressed using agrobacterium in leaf tissues and total proteins were immunoprecipitated at 60 hpi using anti-GFP beads. Input and immunoprecipitated protein (IP) were analyzed by immunoblot analysis with anti-GFP and anti-myc tag antibodies. **(E,F)** LCI assay of the interaction between NbATG5 and p2 in *Nicotiana benthamiana* **(E)**. Luminescence was measured **(F)** in the infiltrated zones expressing nLUC-NbATG5 and cLUC-p2, nLUC-NbATG5 and cLUC-pGUS, nLUC-NbATG5 and cLUC-NbATG12, nLUC-NbATG5 and cLUC-CP, or cLUC-p2 and nLUC-pGUS. Bars indicate standard error from three individual experiments. The asterisks indicate significant difference by Student’s *t* test (^**^p < 0.01).

Since viral proteins from different plant viruses are known to interact with ATGs, the key components of autophagy (Nakahara et al., 2012; Hafren et al., 2017, 2018; Haxim et al., 2017; Li et al., 2018, 2020b), we screened for interactions between the RSV-encoded proteins, p2, pc2, p3, CP and p4, and the ATGs from *Nicotiana benthamiana* (NbATG3, NbATG5, NbATG6, NbATG7 and NbATG8) by bimolecular fluorescence complementation (BiFC) assay. Fluorescence was observed in cells expressing *N. benthamiana* ATG5 (NbATG5) and RSV p2 protein (Figure 1A; Supplementary Figure S1) but not in cells expressing any other combination of viral protein and ATG (Supplementary Figure S1). The interaction between ATG5 and p2 was then further confirmed by yeast two hybrid (Y2H), coimmunoprecipitation (Co-IP) and firefly luciferase complementation imaging (LCI) assays (Figures 1B–F). It was further shown that NbATG5 and p2 colocalized in epidermal cells of *N. benthamiana* (Supplementary Figure S2). Rice ATG5 (*Os*ATG5) also interacted with RSV p2 protein, indicating that the ATG5-p2 interaction was conserved in RSV-infected rice and *N. benthamiana* (Supplementary Figure S3).

### p2 is targeted for degradation by autophagy

**Figure 2:**
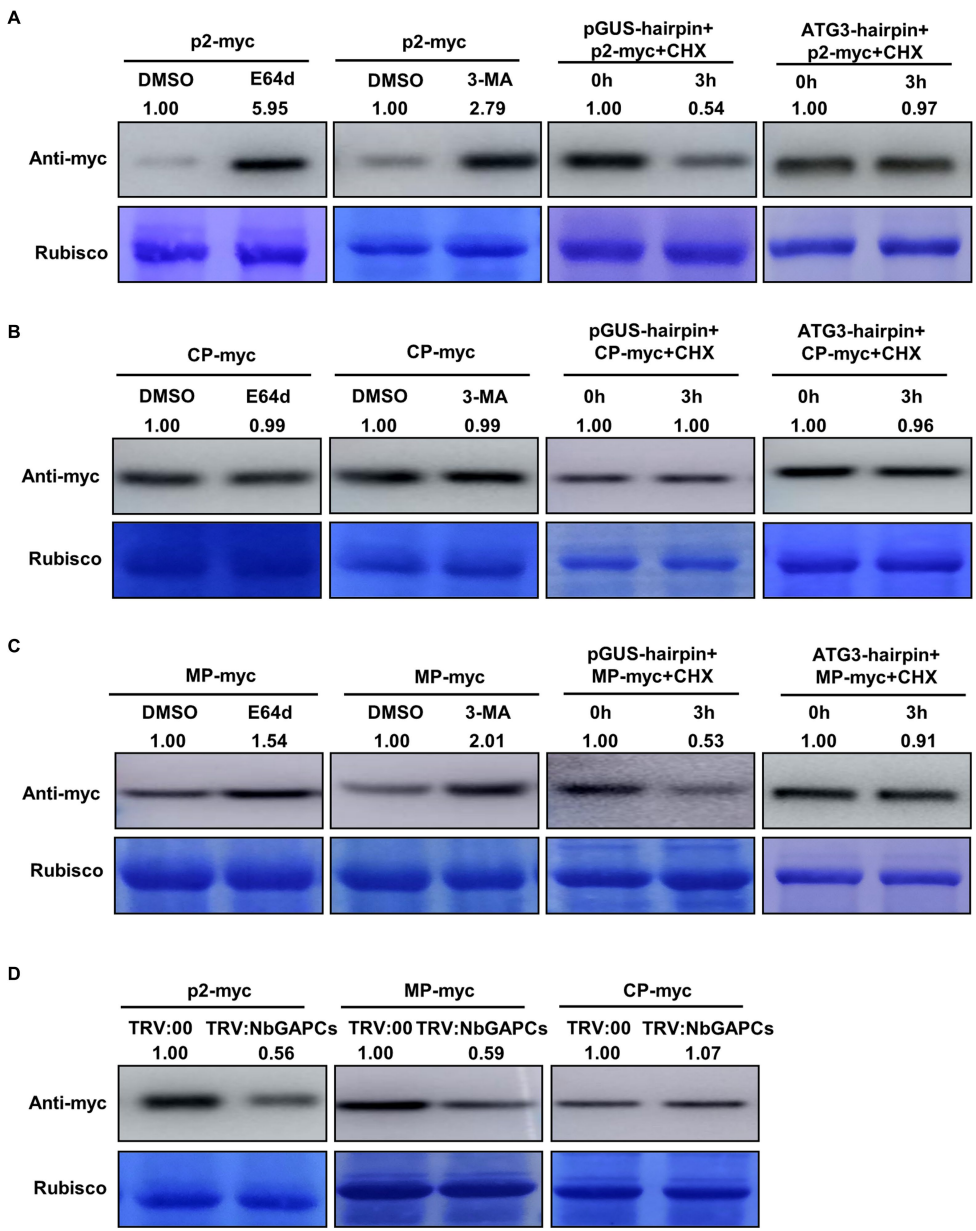
p2 protein is targeted by autophagy for degradation. **(A)** Treatment with the autophagy inhibitors, E-64d and 3-MA, increased the accumulation of RFP-fused p2 protein (p2-myc). Leaves expressing p2-myc by agroinfiltration at 48 hpi were further treated with DMSO, E-64d or 3-MA for 12 h. Total proteins were then extracted for Western blot. Rubisco large subunit was used as a loading control. Silencing of *NbATG3* blocked the degradation of p2. Accumulation of p2-myc was reduced at 3 h post infiltration of CHX in the control leaves expressing GUS-hairpin, indicating the degradation of p2-myc (left panel), while it was not in leaves expressing NbATG3-hairpin. **(B)** Results using RSV coat protein (CP) as the negative control showing that it was not targeted by autophagy for degradation. **(C)** Results using RSV movement protein (MP) as the positive control since this protein has been reported to be degraded through autophagy. **(D)** Silencing of *NbGAPCs* reduced the accumulation of p2. Accumulation of p2-myc was reduced in plants infected with TRV:NbGAPCs compared to that in the control (TRV:00-infected) plants. Two typical replicates in each experiment are shown. Band intensity in blots was calculated by ImageJ from at least three replicates.

The interaction between ATG5 and p2 prompted us to explore whether p2 could be degraded by autophagy. To investigate this, we expressed red fluorescence protein (RFP)-fused p2 (p2-RFP) in leaves of *N. benthamiana* by agroinfiltration and detected the accumulation of p2-RFP protein under treatment with the autophagy inhibitors E-64d and 3-methyladenine (3-MA). E-64d is a cysteine protease inhibitor which prevents the formation of autophagic compartments for degradation when autophagic cargos are released into the vacuolar lumen (Moriyasu and Ohsumi, 1996). 3-MA targets the Type III PI3K to inhibit the induction of autophagy (Seglen and Gordon, 1982). p2-RFP was first expressed in leaves for 48 h and then leaves were infiltrated with E-64d (100 μM), 3-MA (5 mM) or DMSO (control). The accumulation of p2-RFP in the infiltrated leaf area was tested 12 h later by western blot using an antibody to RFP. In leaf area treated with E-64d or 3-MA, p2-RFP accumulated at a higher level than in the control zones expressing the empty RFP (Figure 2A). In the control experiment, the accumulation of empty RFP was not affected significantly by either 3-MA or E-64d (Supplementary Figure S4). We also used RSV CP, that did not interact with ATG5, as a control to detect its accumulation under treatment. CP accumulation was not affected by either E-64d or 3-MA treatment (Figure 2B). Meanwhile, the degradation of RSV MP, that was reported to be targeted by autophagy, was inhibited by E-64d or 3-MA treatment (Figure 2C). These results suggest that p2 may be degraded through autophagy.

To further confirm this hypothesis, we then silenced the *ATG3* gene to inhibit the autophagy pathway and determined whether the degradation of p2 was alleviated. To silence the *ATG3* gene, a plasmid expressing a hairpin RNA of NbATG3 was developed (pNbATG3-hairpin). A control construct expressing the hairpin RNA of an unrelated gene, GUS (β-glucuronidase), was also developed (pGUS-hairpin). The constructs were expressed individually with p2-RFP in leaves by agroinfiltration. The construct expressing hairpin RNAs of *NbATG3* caused the silencing of *NbATG3*, but did not affect the expression of other *NbATGs*, indicating the specific silencing of *NbATG3* in the experiment (Supplementary Figure S5A). Moreover, in leaves expressing ATG3-hairpin, the accumulation of the type II of ATG8 was reduced, indicating that the silencing of *NbATG3* inhibited autophagy as expected (Supplementary Figure S5B). For analysis, at 60 h post infiltration (hpi), the protein synthesis inhibitor cycloheximide (CHX) was infiltrated into leaves to block protein translation. The accumulation of p2-RFP in samples was detected with the monoclonal antibody against RFP at 0 and 3 hpi of CHX. The accumulation of p2-RFP in leaves expressing pGUS-hairpin was reduced at 3 hpi of CHX, indicating the degradation of p2-RFP (Figure 2A). Meanwhile, in leaves expressing the hairpin of *NbATG3*, p2-RFP accumulation was not changed significantly between 0 and 3 hpi, showing that p2-RFP degradation had been inhibited (Figure 2A). In this experiment, RSV CP and MP were also included. The accumulation of CP was not affected by silencing of *NbATG3* but the accumulation of MP, that is reported to be targeted by autophagy, was recovered by silencing of *NbATG3* (Figures 2B,C). The results support the notion that p2 can be degraded through autophagy.

It has been reported that the cytosolic glyceraldehyde-3-phosphate dehydrogenases (GAPCs) regulate autophagy negatively and that silencing of *GAPCs* activates autophagy (Han et al., 2015). To determine whether the activated autophagy could accelerate the degradation of p2, we therefore silenced *GAPCs* using tobacco rattle virus (TRV)-induced gene silencing (VIGS) and detected its effect on p2 accumulation. The accumulation of p2-RFP was significantly less in leaves of *GAPCs-*silenced plants than in the leaves of non-silenced (TRV:00-infected) plants (Figure 2D; Supplementary Figure S5C). Consistently, the positive control RFP-fused MP also accumulated less in *GAPCs-*silenced plants, but RFP-fused CP was not affected (Figure 2D). To further investigate the effect of autophagy on p2 degradation, we treated plants with salicylic acid (SA) to induce autophagy and examined the accumulation of p2. Plants with SA-induced autophagy had decreased accumulation of p2 compared to the control (Supplementary Figure S6).

Taken together, these results demonstrate that RSV p2 protein is targeted by autophagy for degradation.

### Overexpression of *NbATG5* promotes the autophagic degradation of p2

**Figure 3:**
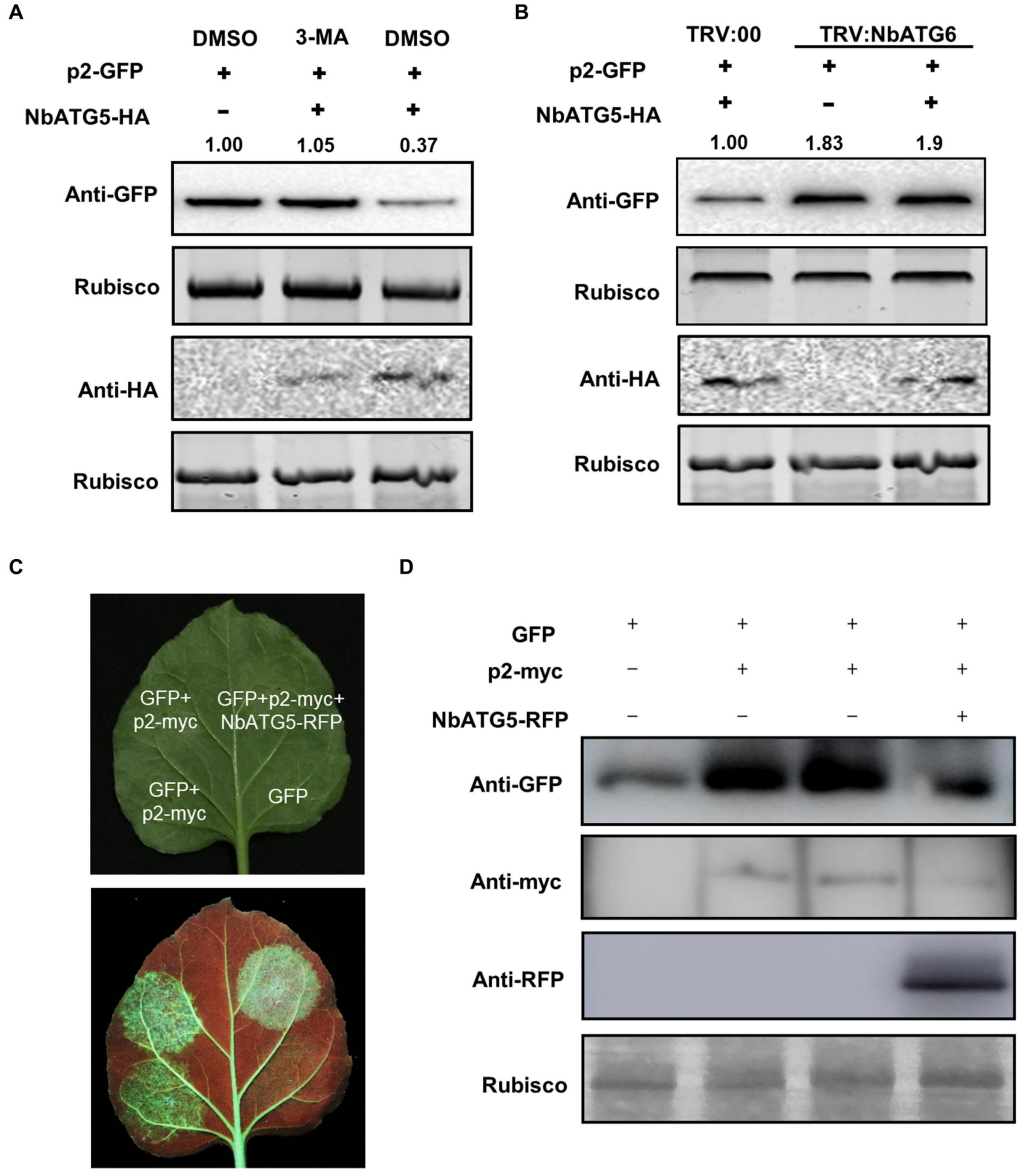
Overexpression of NbATG5 promotes the autophagic degradation of p2. **(A)** Overexpression of NbATG5 reduced the accumulation of p2, while treatment with the autophagy inhibitor 3-MA rescued such reduction. Total proteins were isolated from plant leaves agroinfiltrated with p2-GFP, with empty vector or with NbATG5-HA followed by DMSO or 3-MA treatment. Rubisco large subunit was used as a loading control. The relative level of p2-GFP was calculated by setting control (DMSO) values to 1.0 by ImageJ. **(B)** Silencing of *NbATG6* inhibited NbATG5-mediated degradation of p2-GFP. Plants inoculated with TRV:00 or TRV-Nb*ATG6* at 14 dpi were infiltrated with p2-GFP alone or with NbATG5-HA. Total protein was extracted from infiltrated leaves at 60 hpi. Western blotting assay of the sample protein used anti-GFP or HA. Rubisco large subunit was used as a loading control. The relative level of p2-GFP was calculated by setting the control (TRV:00) values to 1.0 by ImageJ. **(C,D)** Activity of p2 as suppressor of RNA silencing was impaired by overexpression of NbATG5 in *N. benthamiana* 16c plants. GFP fluorescence was revealed by UV illumination at 6 days post infiltration with *Agrobacterium* constructs **(C)**. Immunoblot analysis of protein expression using anti-myc, anti-RFP or anti-GFP antibodies **(D)**. Rubisco large subunit was used as a loading control.

In our recent report, overexpression of *NbATG5* induced autophagy (Zhang et al., 2021b). Since NbATG5 interacted with p2, we wondered whether NbATG5 also had a role in the degradation of p2. We therefore co-expressed NbATG5-HA and p2-GFP in leaves by agroinfiltration and estimated the accumulation of p2 at 60 hpi, using the empty vector with p2-GFP as a control. There was significantly less p2-GFP in leaves expressing NbATG5 than in the controls, whereas NbATG5 expression had no effect on the accumulation of empty GFP or the control RSV CP, indicating that overexpression of NbATG5 accelerated the degradation of p2 (Figure 3A; Supplementary Figures S7A,B). When the experiment was repeated using TRV: ATG6-infected plants, the accumulation of p2-GFP in leaves expressing NbATG5-HA was similar to that in control leaves (Figure 3B; Supplementary Figure S7C). The results support the hypothesis that overexpression of NbATG5 promotes the autophagic degradation of p2. In further experiments, the accumulation of NbATG5 was not affected by expression of p2 (Supplementary Figure S8).

p2 has been identified as a weak suppressor of RNA silencing (Du et al., 2011). As expected, the activity of p2 as the suppressor was impaired by expression of NbATG5 in *N. benthamiana* 16c line (Figures 3C,D).

### Expression of p2 activates autophagy

**Figure 4:**
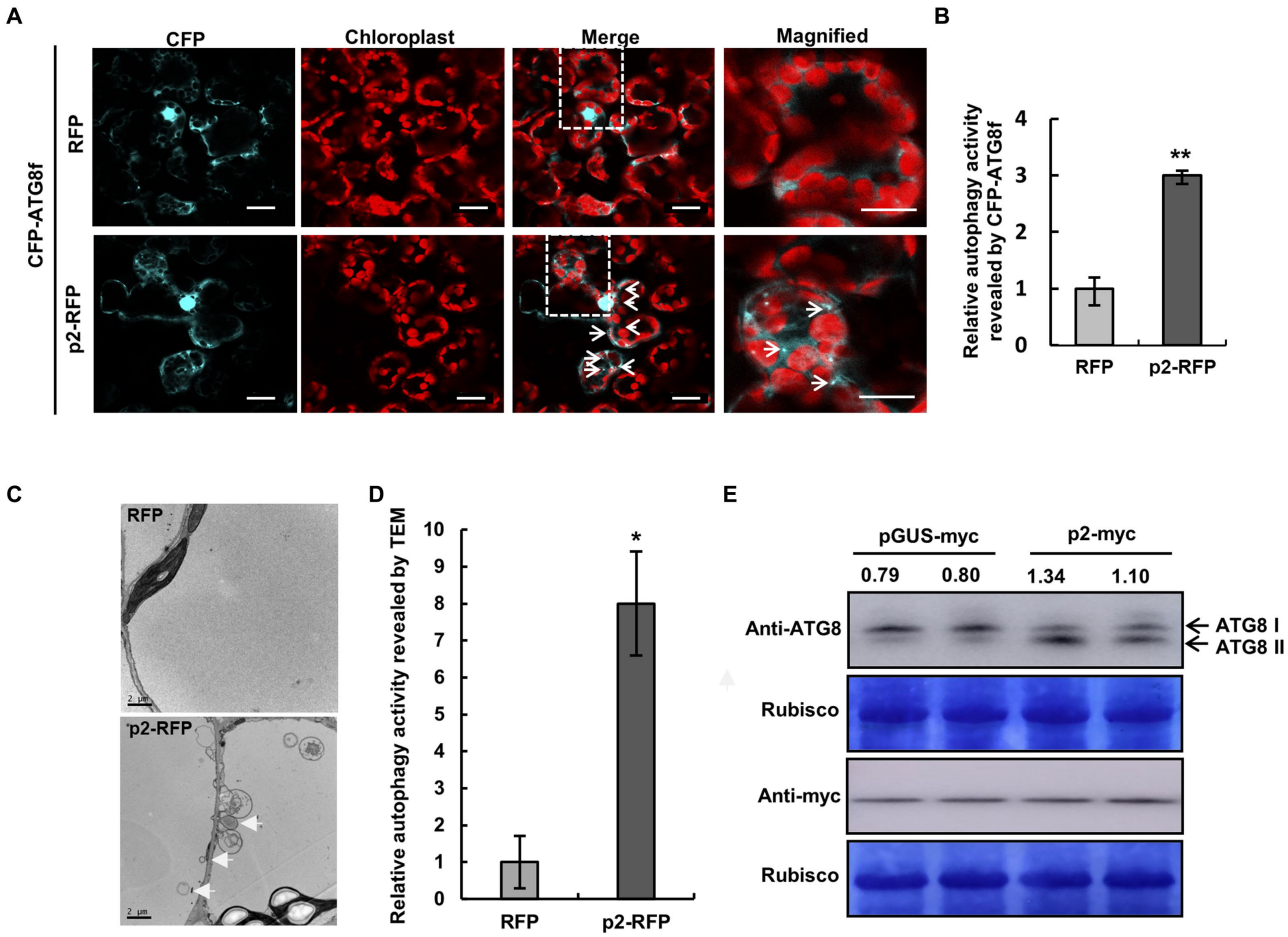
Expression of p2 activates autophagy. **(A)** Representative confocal images of dynamic autophagic activity as revealed by CFP-NbATG8f in control (RFP) and p2-RFP treatments. White arrows show autophagosomes and autophagic bodies labeled by CFP-NbATG8f in cyan in mesophyll cells. Chlorophyll autofluorescence is red. Numerous autophagosomes and autophagic bodies were observed in p2-RFP, but fewer were observed in RFP. Samples were treated with E-64d. Bars, 10 μm. **(B)** Quantification of the autophagy activity shown in **(A)**. Autophagic foci reflected the autophagic activity by CFP-NbATG8f. Relative autophagic activity in cells was normalized to control RFP (which was set to 1.0). Over 150 cells per treatment were used for quantification. Bars indicate standard error from three individual experiments. The asterisks indicate significant difference by Student’s t test (^**^p < 0.01) compared to the control. **(C,D)** TEM analysis of *N. benthamiana* cells inoculated with RFP or p2-RFP **(C)**. White arrows indicate autophagosomes. Relative autophagic activity in cells normalized to control RFP (which was set to 1.0) **(D)**. Approximately 20 cells were used to quantify autophagic structures in each treatment. Experiments were repeated three times. Asterisks indicate significant differences by Student’s t-test compared to control inoculation (*p<0.05). **(E)** Western blotting analysis of type I and type II of NbATG8 in *N*. *benthamiana* leaves expressing pGUS-myc or p2-myc using anti-ATG8 antibodies. The myc-fused partial GUS (pGUS) protein with same number of amino acids as p2 was used as control. The bands intensity was quantified with the ImageJ, and the typeII/typeI ratio is shown.

Next, we investigated whether p2 could affect autophagy. p2-RFP and empty RFP were expressed in leaves of *N. benthamiana* by agroinfiltration, and autophagic activity was monitored using cyan fluorescent protein-tagged NbATG8f (CFP-NbATG8f) as described before (Han et al., 2015). At 60 hpi of co-expression of CFP-NbATG8f and p2-RFP or RFP, there was a diffuse CFP fluorescent signal in cells expressing RFP (Figure 4A; Supplementary Figure S9), but in cells expressing p2-RFP there were many fluorescent foci, indicating that autophagy had been activated (Figures 4A,B). It was also shown that p2 colocalized with autophagosomes receptor ATG8f in epidermal cells of *N. benthamiana* (Supplementary Figure S2). Moreover, multiple autophagic structures could be seen in cells expressing p2-RFP by transmission electron microscopy (TEM; Figures 4C,D). In cells expressing p2, we detected type II ATG8 by Western Blot with an anti-ATG8 antibody, which provides molecular evidence supporting the activation of autophagy by p2 expression (Figure 4E). We also used RSV CP as a control in the analysis and found that CP expression did not increase the number of autophagosomes or accumulation of type II ATG8 (Supplementary Figure S10). Additionally, we investigated the autophagic flux (Dagdas et al., 2016). In the absence of E-64d, the GFP:GFP-ATG8 ratio in leaves containing p2-myc was greater than in leaves containing pGUS-myc (Supplementary Figure S11A). The ratio of GFP:GFP-ATG8 in p2-myc samples treated with E-64d was also higher than that in p2-myc samples without E-64d treatment. In addition, CFP-NbATG8f fluorescence foci increased significantly in the presence of E-64d (Supplementary Figures S11B,C). These results further support the conclusion that expression of p2-myc induces autophagy. In further tests, the expression of *NbATGs* was significantly upregulated by p2, providing additional evidence that p2 activates autophagy (Supplementary Figure S12).

### NbATG5 has a stronger affinity for p2 than for NbeIF4A

**Figure 5:**
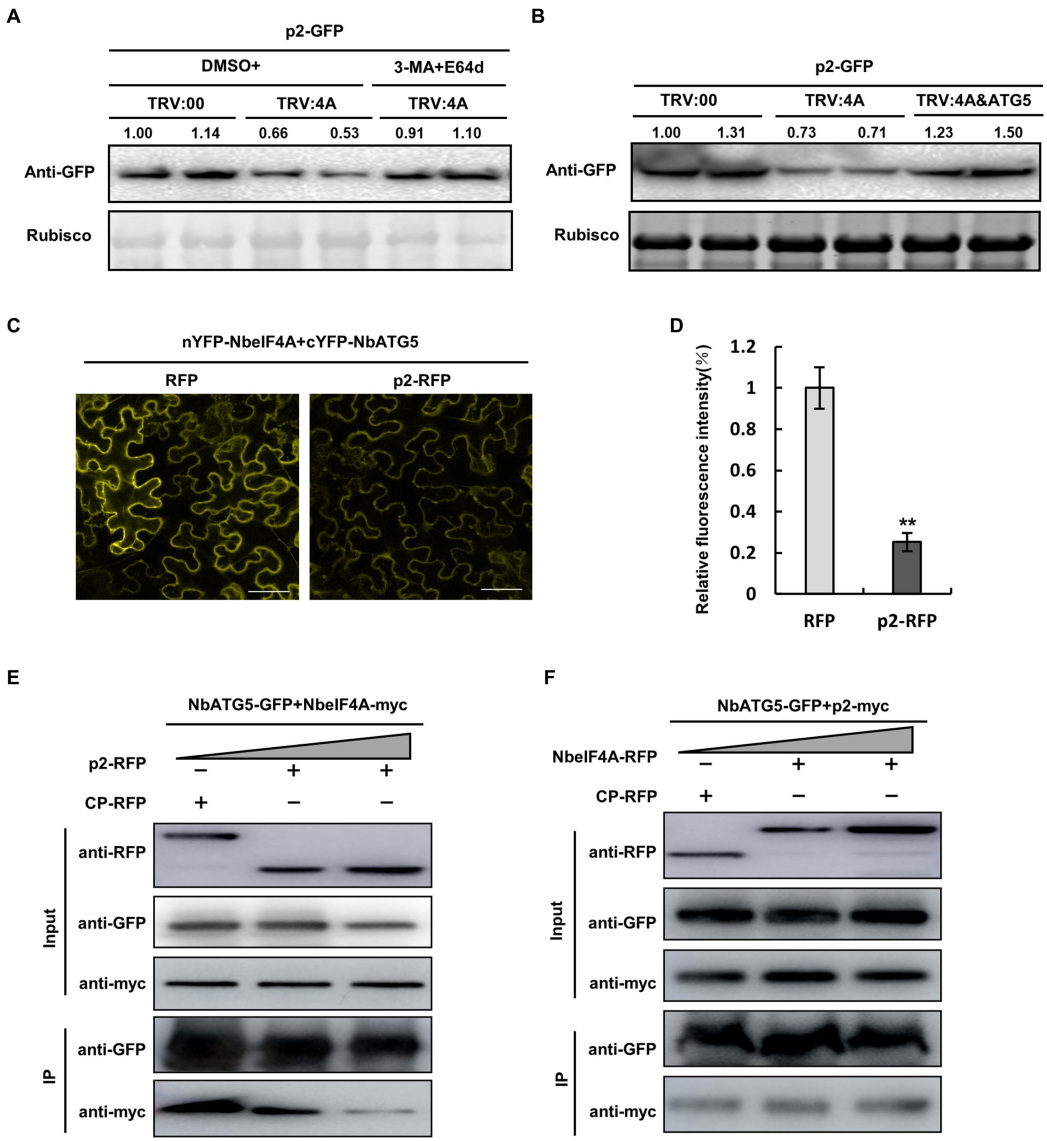
NbATG5 has greater affinity to p2 than to NbeIF4A. **(A,B)** Accumulation of GFP-fused p2 was reduced in *NbeIF4A*-silenced (TRV:4A-infected) plants, compared to that in the control (TRV:00-infected). Meanwhile, the reduction was inhibited by 3-MA or E-64d treatment **(A)** or by co-silencing of ATG5 **(B)**. **(C,D)** BiFC assay showing the interaction between NbeIF4A and NbATG5 with obvious yellow fluorescence in the presence of RFP **(C)**. When p2-RFP was co-expressed, the intensity of yellow fluorescence was significantly reduced **(D)**, indicating that p2 interferes with the interaction between NbeIF4A and NbATG5. Bars, 50 μm. Samples were observed by laser confocal microscopy at 60 hpi. The asterisks indicate significant difference by Student’s *t* test (^**^p < 0.01). **(E,F)** Co-IP assay of NbeIF4A-myc and NbATG5-GFP in the presence of p2; the amount of NbeIF4A immunoprecipitated by the myc antibody was significantly reduced **(E)**. NbeIF4A expression had no effect on the interaction between NbATG5 and p2 **(F)**. At 60 hpi, total proteins were extracted. Rubisco large subunit was used as a loading control. Band intensity in blots was calculated by ImageJ from at least three replicates.

We recently reported that NbeIF4A functions as a negative factor regulating autophagy by interacting with NbATG5 and inhibiting its roles in autophagy (Zhang et al., 2021b). As expected, silencing of *NbeIF4A* accelerated p2 degradation, while treatment with 3-MA and E-64d blocked such acceleration (Figure 5A; Supplementary Figure S13A) and co-silencing of *NbATG5* and *NbeIF4A* inhibited p2 degradation (Figure 5B; Supplementary Figure S13B). Because we had found that p2 interacts with NbATG5, we wondered whether p2 interferes with the interaction between NbeIF4A and NbATG5, which would impair the negative role of NbeIF4A on NbATG5 and thus activate autophagy. We therefore analyzed the interaction between NbeIF4A and NbATG5 in the presence or absence of p2 by BiFC and Co-IP assays. In the BiFC assay, the interaction between NbeIF4A and NbATG5 was shown by obvious yellow fluorescence in the presence of RFP, but when p2-RFP was co-expressed, the intensity of yellow fluorescence was significantly reduced, showing that p2 interferes with the interaction between NbeIF4A and NbATG5 (Figures 5C,D; Supplementary Figure S14). Consistently, results of the Co-IP assay showed that with increasing amounts of p2, the amount of NbeIF4A immunoprecipitated by antibody was significantly reduced (Figure 5E). However, the presence of NbeIF4A had no effect on the interaction between NbATG5 and p2 (Figure 5F). These results indicate that the interaction between NbATG5 and p2 is more stable than that between NbATG5 and NbeIF4A.

## Discussion

Autophagy is being recognized as an immune pathway against viruses in plants (Yang et al., 2020; Ismayil et al., 2020b). Many diverse viruses, including CaMV, TuMV, CMV and geminiviruses, are now known to be regulated negatively by autophagy as their viral proteins are degraded (Nakahara et al., 2012; Hafren et al., 2017, 2018; Haxim et al., 2017; Li et al., 2018, 2020b). Two different viral proteins of TuMV are targeted by autophagy to be degraded (Hafren et al., 2018; Li et al., 2018), but there are few viruses in which two or more viral proteins are reported to be targeted in this way. In our previous reports, we demonstrated that autophagy and the 26S proteosome pathway play defense roles against RSV by recognizing the p3 protein for degradation (Chen et al., 2020; Jiang et al., 2021). Recently, the MP of RSV has also been shown to be targeted by autophagy for degradation during RSV infection (Li C. et al., 2021). Here, we showed that RSV p2 could also be degraded by autophagy, thus identifying a third RSV protein that is targeted by autophagy (Figure 2). This finding underlines the essential role of autophagy in defense against RSV during the balance of virus-plant interactions.

Autophagy can be induced in plants infected by CLCuMuB, TuMV or RSV, but the mechanism by which viruses induce autophagy is not yet well understood (Haxim et al., 2017; Li et al., 2018; Jiang et al., 2021). Ismayil et al. (2020b) recently identified a possible mechanism by which CLCuMuB induces autophagy. βC1 encoded by CLCuMuB interacts with the negative autophagic regulator GAPC, blocking its interaction with ATG3, hence inducing autophagy in *N. benthamiana* (Han et al., 2015; Ismayil et al., 2020a). We recently found that NbeIF4A functions as a negative regulator of autophagy by interacting with and inhibiting ATG5, while its transcript can be targeted for silencing by a vsiRNA, indicating one way used by viruses to induce autophagy (Zhang et al., 2021b). Interestingly, the results here showed that p2 expression induced autophagy and that ATG5 interacted preferentially with p2 rather than with eIF4A, which suggests that autophagy may be induced by p2 that releases ATG5 from the inhibition of eIF4A (Figure 5). It is also possible that p2 targets the ATG5/eIF4A complex itself as part of a pro-viral response or that autophagy is recruited during a certain infection stage to limit and/or balance p2 levels with other viral proteins to increase plant tolerance to infection. These possibilities need exploration in the context of viral infection. This has not been attempted here because it would be difficult to exclude the induction of autophagy by vsiRNA. An ideal experiment using a viral clone would provide further evidence. Minireplicon of RSV have been reported, which provides a glimmer of hope for such research (Feng et al., 2021; Zhang et al., 2021a).

Evidence from various studies also suggests that autophagy can be manipulated or even exploited by viruses to degrade the components in plant immunity, hence benefiting the virus infection, which highlights the complex roles of autophagy in plant–virus interactions (Derrien et al., 2012; Cheng and Wang, 2017; Hofius et al., 2017; Li et al., 2017, 2020a; Huang et al., 2019; Yang et al., 2020; Ismayil et al., 2020b). In fact, during RSV infection, autophagy is also reported to contribute to RSV movement, suggesting that the interaction between autophagy and RSV is more complicated than what we have shown (Fu et al., 2018). Recently, the γb protein of barley stripe mosaic virus (BSMV) was shown to competitively interfere with the interaction between ATG7 and ATG8 to subvert autophagy and promote viral infection (Yang et al., 2018). It will be interesting next to investigate whether RSV has a counter strategy to fight back against autophagy. All the factors participating in the interaction between autophagy and RSV need to be identified to help further dissect the mechanisms by which RSV regulates autophagy.

## Materials and methods

### Plant growth conditions and viral inoculation

*Nicotiana benthamiana* plants were grown in pots at 24°C and 60% relative humidity under a 16 h-light/8 h-dark cycle. Plants at 2-weeks old were used for *Agrobacterium* infiltration. pTRV1 and pTRV2 or its derivatives were introduced into Agrobacterium (*Agrobacterium tumefaciens*) strain GV3101 for VIGS assays in infiltration buffer [10 mM MgCl_2_, 10 mM MES (pH 5.6), and 100 μM acetosyringone] as described before (Jiang et al., 2021). For co-silencing of genes, bacterial suspensions were mixed in a 1:1 ratio.

### Plasmid constructs

Rice stripe virus p2 was amplified by PCR using cloned cDNA as template and the resulting PCR products were cloned into the pJG045 vector. The DNA fragments for p2-RFP, p2-GFP, p2-myc, cLUC-p2, nYFP-p2 were obtained by overlapping PCR. *NbATG5* cDNA was amplified by PCR and cloned into the pJG045 vector. DNA fragments for NbATG5-GFP, NbATG5-RFP, cYFP-NbATG5, and nLUC-NbATG5 were obtained by overlapping PCR. The PCR products of *p2* or *NbATG5* were cloned into pGADT7 or pGBKT7 to construct AD-p2 and BK-*NbATG5* by overlapping PCR. The full-length *NbATG3*, *NbATG6*, *NbATG7*, *NbATG8f*, *NbATG12*, *RSV P3*, *P4*, *PC2* and *OsATG5* were cloned as described previously (Chen et al., 2020; Jiang et al., 2021; Zhang et al., 2021b).

*NbeIF4A* cDNA was amplified by PCR and cloned into the pJG045 vector. The DNA fragments for nYFP-NbeIF4A, NbeIF4A-myc, NbeIF4A-RFP were obtained by overlapping PCR. RSV CP and MP were amplified by PCR using cloned cDNA as template and the resulting PCR products were cloned into the pJG045 vector. The DNA fragments for CP-myc, cLUC-CP, nYFP-CP, and MP-myc, cLUC-MP, nYFP-MP were obtained by overlapping PCR as described previously (Han et al., 2020).

To construct a TRV-based recombinant VIGS vector containing *NbeIF4A* or *NbATG6*, a partial fragment of each gene was amplified by PCR using the appropriate primer pair and cloned into the pTRV2-lic vector. Two fusion DNA fragments for *NbeIF4A* and *NbATG6* were obtained by overlapping PCR and cloned into the pTRV2-lic vector.

To construct RNAi vectors targeting *GUS*, *NbATG3, NbATG5, NbATG6, NbATG7* and *NbATG8f*, a partial fragment of each gene was amplified by PCR using the appropriate primer pair and cloned into the hairpin vector.

The primers used for these vectors are listed in Supplementary Table 1.

### Y2H, BiFC, and LCI

Y2H and BiFC assays were performed as described previously (Jiang et al., 2021). Fluorescence was examined by confocal microscopy (Leica TCS SP8, Mannheim, Germany). For LCI assays, we transiently expressed the combinations in *N. benthamiana* by Agrobacterium-mediated infiltration. The infiltrated leaves were kept in the dark for 24 hpi and then treated with white light for 48 hpi. The leaves were detached at 72 hpi and sprayed with 1 mM luciferin. After keeping the materials in the dark at 28°C for 5 min to quench the chlorophyll auto-fluorescence, we collected the luciferase signal with a low-light cooled CCD imaging apparatus (Amersham Image 680).

### RNA extraction and RT-qPCR

Total RNA was isolated from the infiltrated leaves by the Trizol reagent and treated with RNase-free DNase I (TaKaRa, Japan) to remove potential DNA contamination. RT-qPCR analysis was conducted and analyzed as described previously (Jiang et al., 2021). Primers used for RT-qPCR are listed in Supplementary Table 1.

### Chemical treatments and immunoblotting

3-MA (5 mM) and E-64d (100 μM) dissolved in DMSO were used to inhibit the autophagy pathway. These chemical reagents (or DMSO as control) were infiltrated into leaves and samples were collected at 8–12 hpi for western blot analysis. We performed immunoblotting with anti-myc (Sigma, St. Louis, United States), anti-RFP (Sigma, St. Louis, United States), anti-HA (Sigma, St. Louis, United States) or anti-GFP (Sigma, St. Louis, United States) monoclonal antibodies,

anti-ATG8 (Agrisera, Sweden) polyclonal antibody, and anti-monoclonal or anti-polyclonal (Sigma, St. Louis, United States) secondary antibody at 1:10,000 dilution.

### Confocal microscopy and transmission electron microscopy (TEM)

For confocal microscopy (Leica TCS SP8, Mannheim, Germany), the fluorescent proteins were excited by the LD laser line for GFP (488 nm), CFP (405 nm), and YFP (514 nm). Detection bands were optimized for each fluorophore group to avoid emission bleeding.

For electron microscopy, leaves were cut into small fragments (1–2 mm^2^) and fixed by infiltration with 0.1 M PBS buffer containing 2.5% glutaraldehyde. Samples were post-fixed in 2% OsO_4_, followed by dehydration in ethanol and acetone, before embedding in Spurr resin (SPI Supplies). Sections were cut with a diamond knife on an ultramicrotome (EM UC7; Leica, Germany) and collected, on copper grids double stained with uranyl acetate and lead citrate before examination.

### Statistical analysis

The intensity of the protein bands detected by protein gel blot analysis and the fluorescence intensities were quantified using the ImageJ. Statistical analysis on the numbers of autophagosomes using SPSS software. The data were compared using one-way analysis of variance (ANOVA). The numbers of autophagosomes were analyzed by Students’ *t*-test.

### Accession numbers

GenBank accession numbers of genes and viral sequences analyzed in this study are as follows: *NbeIF4A* (XM019409915), *NbATG2* (KU561373), *NbATG3* (KX369396), *NbATG5* (KX369397), *NbATG6* (AY701316), *NbATG7* (KX369398), *NbPI3K* (KX120977), *NbATG8f* (KU561372), *NbATG9* (KX369399), *NbJoka2*(018766878), RSV *p2* (DQ333943), *NbActin* (AY179605).

## Data availability statement

The original contributions presented in the study are included in the article/Supplementary material, further inquiries can be directed to the corresponding authors.

## Author contributions

FY, XZ, and YW contributed to conception and design of the study. XZ organized the database. XZ, QW, PR, YL, and ZS performed the statistical analysis. XZ and FY wrote the first draft of the manuscript. XZ, FY, YL, and JC wrote sections of the manuscript. All authors contributed to the article and approved the submitted version.

## Funding

This work was supported by the National Natural Science Foundation of China (U22A20479) and the K. C. Wong Magna Fund of Ningbo University.

## Conflict of interest

The authors declare that the research was conducted in the absence of any commercial or financial relationships that could be construed as a potential conflict of interest.

## Publisher’s note

All claims expressed in this article are solely those of the authors and do not necessarily represent those of their affiliated organizations, or those of the publisher, the editors and the reviewers. Any product that may be evaluated in this article, or claim that may be made by its manufacturer, is not guaranteed or endorsed by the publisher.
